# The Brief Case: Adjudication of discrepant genotypic and phenotypic antimicrobial susceptibility testing results in a patient with *Staphylococcus aureus* bacteremia

**DOI:** 10.1128/jcm.00809-25

**Published:** 2026-02-11

**Authors:** Fabiola Reyes Curcio, Justin McCallum, Gina Borrelli, Beverley Orr, Zoe Weiss, E. Zachary Nussbaum

**Affiliations:** 1 Department of Pathology and Laboratory Medicine, Tufts Medical Center 1867, Boston, Massachusetts, USA; 2 Department of Geographic Medicine and Infectious Disease, Tufts Medical Center 1867, Boston, Massachusetts, USA; Endeavor Health, Evanston, Illinois, USA

**Keywords:** MRSA, antimicrobial susceptibility

## CASE

A 41-year-old female with ongoing injection drug use and recent methicillin-susceptible *Staphylococcus aureus* (MSSA) bacteremia and lumbar epidural abscess requiring operative washout presented with lumbar wound dehiscence. The patient was taken back to the operating room for surgical debridement of the paraspinal infection and placement of extensive hardware for spinal stabilization. Operative cultures again grew MSSA. Two sets of peripheral blood cultures were obtained on admission. Three of four bottles turned positive, with Gram stain revealing gram-positive cocci in clusters in all three positive bottles. Multiplex PCR testing was performed directly on a positive blood specimen using the BioFire FilmArray blood culture identification 2 (BCID2) panel (bioMérieux, Marcy l’Etoile, France). Results were positive for *S. aureus* and *Staphylococcus epidermidis*. Both *mecA/C* and *mecA/C* + MREJ targets were detected, and the patient was initiated on vancomycin. Growth on solid media and subsequently matrix-assisted laser desorption/ionization-time of flight analysis confirmed the identification of *S. aureus* in all three bottles, as well as *S. epidermidis* in one bottle—the bottle on which initial BCID2 testing was performed. Since the *mecA/C* + MREJ target is specific for methicillin-resistant *S. aureus* (MRSA), it was expected that phenotypic antimicrobial susceptibility testing (AST) would confirm oxacillin resistance. However, AST performed on five well-isolated colonies of *S. aureus* from a subculture of each positive blood culture bottle using the VITEK 2 instrument demonstrated oxacillin (MIC = 0.5 μg/mL) and cefoxitin susceptibility in all *S. aureus* isolates and resistance only in the *S. epidermidis* isolate. This represented a clinically significant discrepancy between rapid molecular AST and conventional phenotypic AST. Further laboratory investigation was required.

No pre-analytic errors were identified, and isolate purity was confirmed. BCID2 testing was repeated on the same bottle on which it was initially performed, and VITEK 2 testing was repeated on *S. aureus* colonies from each positive bottle. The BCID2 and AST results were identical to the initial testing. Cefoxitin disc diffusion was performed on *S. aureus* isolates from each bottle and yielded susceptible results. Immunochromatographic penicillin-binding protein 2a (PBP2a) testing was performed on *S. aureus* isolates from each bottle (Clearview PBP2a, Abbott Diagnostics) and was negative. BCID2 testing was performed on one of the blood culture samples that had grown only *S. aureus* without growth of *S. epidermidis*. This was positive for *S. aureus* and the *mecA/C* + MREJ target, but the *S. epidermidis* and *mecA/C* (alone) targets were negative, once again suggesting the presence of MRSA.

**Fig 1:**
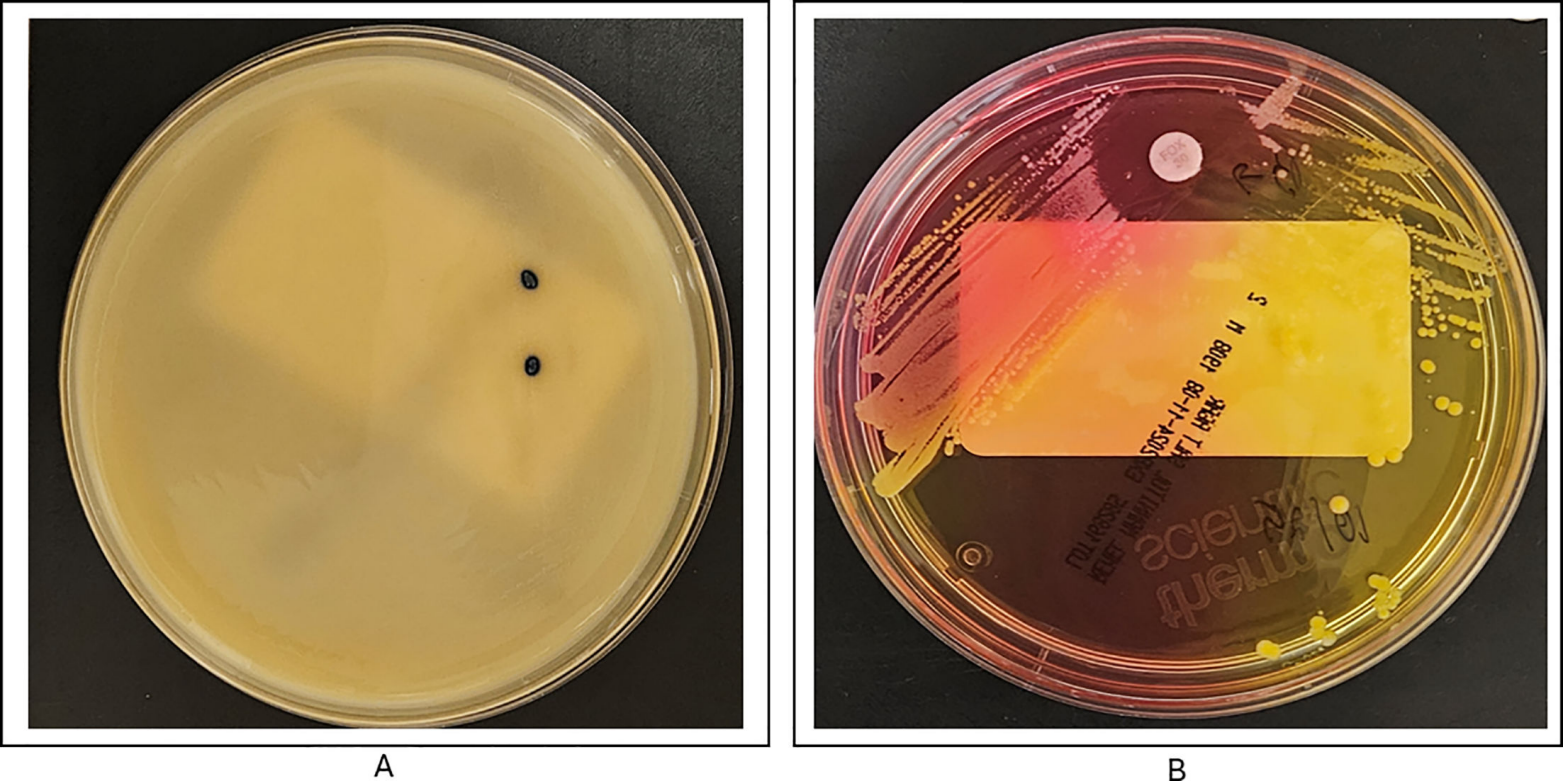
(**A**) MRSA chromogenic agar showing denim blue colonies suggestive of MRSA. (**B**) Mannitol salt agar with a cefoxitin disc. While the disc produces a predominant surrounding zone, several colonies of MRSA are seen within the zone of clearance.

**Fig 2:**
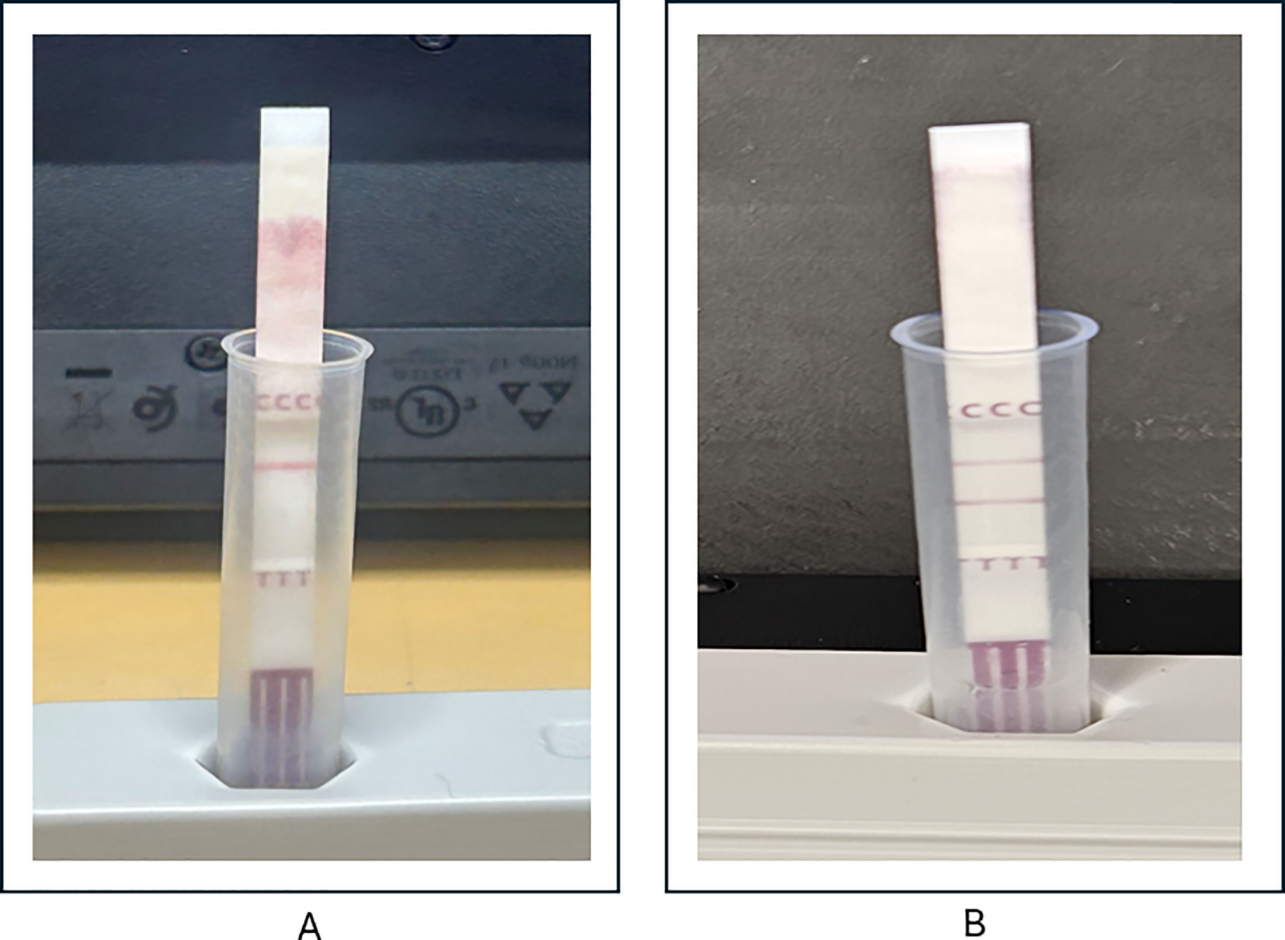
(**A**) Negative PBP2A immunochromatographic test, indicated by a positive control line and negative test line. (**B**) Positive PBP2A test, indicated by positive control and test lines, confirming the production of PBP2a.

To further evaluate the presence of MRSA in the blood culture samples, the primary blood specimens were inoculated onto MRSA selective chromogenic agar (Spectra MRSA medium, Thermo Scientific) and incubated overnight. The following day, two colonies with surrounding blue discoloration were noted, suggestive of MRSA (Fig. 1A). PBP2a testing on these isolates was positive (Fig. 2B), and phenotypic AST revealed oxacillin resistance (MIC > 4 µg/mL). Additionally, blood was inoculated onto mannitol salt agar (Thermo Scientific) and incubated overnight with a cefoxitin disc. The following day, yellow colonies were noted in multiple quadrants of the media, with a clear zone of inhibition around the cefoxitin disc, suggestive of MSSA. However, on careful examination, several pinpoint colonies were noted within the zone of clearance around the cefoxitin disc, suggestive of a small subpopulation of MRSA (Fig. 1B). It was concluded that the patient had bacteremia with both MSSA *and* MRSA, with the MSSA clearly predominating. The *S. epidermidis* was considered a contaminant. Notably, 1 week after the initial blood cultures were obtained, the patient was noted to have an evolving right arm abscess. The culture of this lesion ultimately grew MRSA. This was the likely source of the MRSA bacteremia, while the high-grade MSSA bacteremia was secondary to the epidural abscess.

Although the wound culture that grew MRSA would have ultimately dictated the use of an antibiotic with activity against MRSA, correctly identifying the subpopulation of MRSA in the bloodstream was of profound clinical significance. Particularly in the setting of new spinal hardware, prolonged treatment with vancomycin and subsequently dalbavancin was prescribed. Had only MSSA bacteremia been reported, the antibiotic treatment plan would have likely been significantly different. The successful cultivation of MRSA also allowed for complete AST, including oral agents that could be used for antibiotic suppression in the setting of indwelling hardware.

## DISCUSSION

Detection of antimicrobial resistance still largely relies on conventional culture-based phenotypic AST. However, this is limited by delayed turnaround times, typically of at least 24 h from the time of blood culture positivity, as testing can only be performed on growing isolates and not directly from clinical specimens. There has been increased utilization of rapid molecular antimicrobial resistance testing, which relies on the identification of well-characterized resistance genes and can be performed directly from clinical specimens (1, 2). On occasion, discrepancies between rapid molecular resistance testing and conventional phenotypic AST can occur. Here, we outline a case of such a discrepancy related to antimicrobial resistance in *S. aureus* isolated from blood culture, a clinical scenario of high consequence, and review several methods of laboratory adjudication.

MRSA was first clinically observed in the early 1960s, shortly after the introduction of methicillin, the first semisynthetic penicillinase-resistant β-lactam antibiotic (3, 4). *S. aureus* bacteremia is associated with high mortality rates in excess of 25% (5). Of note, MRSA bacteremia is associated with increased mortality, rates of bacteremia recurrence, and hospital length of stay compared with MSSA (5, 6). As such, it is imperative that laboratories be equipped to quickly identify antimicrobial resistance within *S. aureus* isolates, particularly when isolated from blood culture.

The BioFire FilmArray BCID panel, which has become increasingly utilized in clinical laboratories, combines organism identification with detection of frequently associated resistance genes. It is employed on positive blood cultures, requires minimal hands-on time, and provides results within approximately 1 h. The first BCID panel received FDA approval in 2013 (7). The only staphylococcal resistance target on the original panel was *mecA* alone. However, since *mecA* positivity represents the predominant underlying resistance mechanism to β-lactam antibiotics for both *S. aureus* and staphylococci other than *S. aureus* (SOSA)—by encoding for the altered penicillin-binding protein PBP2a—the detection of *mecA* in blood cultures positive for both *S. aureus* and SOSA was difficult to interpret. It could represent *mecA* associated with SOSA or *S. aureus*, and it was not possible to discern which from this information alone. An updated panel, the BCID2, received FDA clearance in 2020 (8). This panel incorporates an additional target, *mecA/C* + MREJ combined, in addition to the target for *mecA/C* alone. The *mecA/C* + MREJ target identifies a specific sequence at the junction between the *mecA* and *orfX* genes that is specific for MRSA. Only this combined target will be positive in pure MRSA bacteremia, while the *mecA/C* alone target will not trigger a positive result. Conversely, oxacillin-resistant SOSA organisms would produce a positive *mecA/C* target alone with a negative *mecA/C* + MREJ target. This junctional target, in combination with targets for *mecA*, thermostable nuclease, and staphylococcal protein A, is utilized for the specific identification of MRSA in other FDA-cleared blood culture identification assays (9, 10). However, several FDA-cleared assays for molecular detection of resistance in blood culture are only capable of identifying *mecA*, making it challenging to interpret results of polymicrobial cultures mixed with MRSA and oxacillin-resistant SOSA (11, 12). In the clinical case highlighted above, the blood culture was positive for *S. aureus*, *S. epidermidis*, *mecA/C* (alone), and *mecA/C +* MREJ. This would be correctly interpreted as representing the presence of both MRSA and oxacillin-resistant *S. epidermidis*. Therefore, when phenotypic AST revealed MSSA, a major discrepancy was identified. It should be noted that the *mecA/C* alone target will only be reported as positive if associated with *S. epidermidis* or *Staphylococcus lugdunensis*, as these are the only two SOSA organisms identified to the species level on the BCID2 panel.

When such AST discrepancies are identified in *S. aureus*, directly testing isolated colonies for PBP2a production is a rapid and useful benchside tool to assist in adjudication (1, 13–15). PBP2a, the protein product of the *mecA* gene, is an altered penicillin-binding protein that has a low affinity for beta-lactam antibiotics and is responsible for beta-lactam resistance in approximately 99% of MRSA isolates (14). We employed the Abbott Clearview immunochromatographic PBP2a assay, an FDA-cleared, inexpensive, and highly sensitive method that provides results within minutes (15). This assay utilizes recombinant monoclonal antibody fragments to directly detect the PBP2a protein from bacterial isolates grown in culture. When testing was negative on the *S. aureus* blood isolates (Fig. 2A), we considered several other reasons for the discrepancy between genotypic and phenotypic AST results.

One possibility was that the BCID2 was detecting the presence of *mecC*, as opposed to the far more common *mecA*, since *mecC*-mediated resistance is not detected by PBP2a testing. However, this seemed less likely since the isolates demonstrated reproducible susceptibility to cefoxitin, whereas *mecC*-positive strains are typically cefoxitin resistant (16). The possibility of *mecA* dropout was also entertained. This is a well-described phenomenon whereby the MREJ genetic sequence is present but lacks the *mecA* gene (17). In a pure blood culture, *mecA* would not be detected in these mutants, and thus, the *mecA/C* + MREJ target would be negative. However, in a mixed culture with a *mecA*-containing SOSA, *mecA* from the SOSA organism could be detected in addition to the MREJ sequence in the *S. aureus* isolate, thus producing a positive combined *mecA/C* + MREJ result in the absence of MRSA. Additionally, it was possible that the blood culture was even further mixed—containing both MSSA and a very small amount of MRSA that was unable to be isolated in culture. Polymicrobial bacteremia is relatively common in people who inject drugs (18), though combined MRSA and MSSA bacteremia occurs in less than 1% of cases (19).

For further investigation, we returned to the primary specimen and inoculated blood directly onto chromogenic MRSA screening agar (Spectra MRSA medium, Thermo Scientific), as well as mannitol salt agar (Thermo Scientific) with a cefoxitin disc to attempt to detect a small subpopulation of MRSA in the sample. MRSA chromogenic agar contains antibiotics aimed at suppressing the growth of competitor organisms and MSSA. It also contains a chromogen that yields a blue denim color when MRSA is present due to phosphatase activity. This product is approved for use on positive blood cultures with gram-positive cocci on Gram stain. When two blue colonies were detected the following day (Fig. 1A), they were tested for PBP2a production and found to be positive (Fig. 2B). AST on these isolates revealed an oxacillin MIC > 4. This confirmed that there was a very small amount of MRSA in the blood culture, which was unable to be detected on initial subculture due to the overwhelming predominance of MSSA. The production of PBP2a also excluded the possibility that the genotypic results were related to *mecA* dropout. Similarly, Fig. 1B depicts the presence of several colonies within the zone of clearing around a cefoxitin disc on mannitol salt agar, suggestive of a small subpopulation of MRSA. Mannitol salt agar was selected since it is a selective medium used to cultivate the growth of staphylococci. Staphylococci can tolerate the 7.5% NaCl concentration in this medium, which inhibits the growth of most other bacteria (20). In addition, the media can distinguish between *S. aureus* and SOSA based on their ability to ferment mannitol. *S. aureus* can ferment mannitol, which causes the underlying media to appear yellow, whereas other staphylococci are unable to ferment mannitol, and thus, the underlying media remains pink. While use on positive blood culture broth is not specifically mentioned in the package insert, it is intended for use on specimens containing infectious microorganisms and for subculture.

This case illustrates how PBP2a testing, subculture on selective media, and subculture in the presence of a cefoxitin disc can be used to adjudicate discordant genotypic and phenotypic AST results in cases of *S. aureus* bacteremia. Additional testing methods that were not employed in this case but may help resolve such discrepancies include, but are not limited to, *mecA* nucleic acid amplification testing directly from cultured isolates, use of oxacillin NaCl screening agar (13), and whole-genome sequencing (21, 22). Laboratories can access formal guidance for resolving genotypic-phenotypic susceptibility discrepancies related to *S. aureus* bacteremia in Appendix G of the CLSI M100 document (16).

## SELF-ASSESSMENT QUESTIONS

1. Which of the following is the predominant resistance mechanism in MRSA?
  - Extended-spectrum beta-lactamase production
  - The *mecA* gene encodes the production of PBP2a
  - The *vanA* gene encodes the production of cell wall proteins with lower affinity for vancomycin
  - Inducible resistance mediated by the *erm* gene
2. Which of the following genetic targets is specific for MRSA?
  - *mecA/C* + MREJ
  - *mecA/C* alone
  - CTX-M
  - *vanA/B*
3. Where can laboratories access additional guidance on resolving discrepancies between phenotypic and genotypic susceptibility testing in *S. aureus?*
  - The CLSI M45 manual
  - The CLSI M100 (Appendix G)
  - The local antibiogram
  - CLSI guideline documents do not address molecular detection of resistance

## ANSWERS TO SELF-ASSESSMENT QUESTIONS

1. Which of the following is the predominant resistance mechanism in MRSA?
  - Extended-spectrum beta-lactamase production
  - The *mecA* gene encodes the production of PBP2a
  - The *vanA* gene encodes the production of cell wall proteins with lower affinity for vancomycin
  - Inducible resistance mediated by the *erm* gene

Answer: b. The *mecA* gene encodes the altered penicillin-binding protein PBP2a, which has a low affinity for beta-lactam antibiotics. This allows *S. aureus* to continue synthesizing its cell wall even in the presence of these antibiotics, rendering them ineffective. Other mechanisms of resistance mediated by *mecC* and/or hyperproduction of beta-lactamases have been well described but account for a very small minority of cases.

- Which of the following genetic targets is specific for MRSA?
  - *mecA/C* + MREJ
  - *mecA/C* alone
  - CTX-M
  - *vanA/B*

Answer: a. The *mecA/C* + MREJ target identifies a specific sequence at the junction between the *mecA* and *orfx* genes that is specific for MRSA. Only this combined target will be positive in pure MRSA bacteremia. This target will not be positive in cases of bacteremia with oxacillin-resistant staphylococci other than *Staphylococcus aureus*. Only in the setting of a mixed blood culture should both the *mecA* alone and *mecA/C* + MREJ targets be positive.

- Where can laboratories access additional guidance on resolving discrepancies between phenotypic and genotypic susceptibility testing in *S. aureus?*
  - The CLSI M45 manual
  - The CLSI M100 (Appendix G)
  - The local antibiogram
  - CLSI guideline documents do not address molecular detection of resistance

Answer: b. The CLSI M100 document contains guidance on resolving such discrepancies in Appendix G, entitled “Using Molecular Assays for Resistance Detection.” Multiple testing modalities for the detection of resistance in *S. aureus* are highlighted, and guidance is offered on adjudicating scenarios where genotypic and phenotypic susceptibility testing results do not initially align. This is not provided in the M45 manual. While local antibiogram data should indicate the percentage of *S. aureus* isolates identified as MRSA in a given laboratory, guidance on adjudicating laboratory testing discrepancies is generally not provided.

**TAKE HOME POINTS**

- With increasing use of commercially available rapid molecular antimicrobial resistance testing, clinical laboratories may encounter discrepancies between genotypic and phenotypic AST results.
- For molecular detection of resistance in *S. aureus* from blood culture, the *mecA/C* + MREJ combined target is specific for MRSA. Staphylococci other than *S. aureus* that contain the *mecA* gene would not trigger a positive MREJ result.
- PBP2a, the protein product of the *mecA* gene, is an altered penicillin-binding protein that has a low affinity for beta-lactam antibiotics and is responsible for beta-lactam resistance in approximately 99% of MRSA isolates.
- Laboratories can access formal guidance for resolving genotypic-phenotypic susceptibility discrepancies related to *S. aureus* bacteremia in Appendix G of the CLSI M100 document. Use of MRSA chromogenic agar and subculture in the presence of a cefoxitin disc may be useful adjuncts, particularly in the setting of mixed blood cultures. Due to increased sensitivity of molecular methods, and in accordance with CLSI guidance, when discrepancies cannot be definitively resolved, *S. aureus* isolates should be reported as MRSA if genotypic testing is positive for molecular targets of resistance.
